# Nutraceutical Features of the Phycobiliprotein C-Phycocyanin: Evidence from *Arthrospira platensis* (*Spirulina*)

**DOI:** 10.3390/nu16111752

**Published:** 2024-06-03

**Authors:** Valentina Citi, Serenella Torre, Lorenzo Flori, Luca Usai, Nazlim Aktay, Nurhan Turgut Dunford, Giovanni Antonio Lutzu, Paola Nieri

**Affiliations:** 1 Department of Pharmacy, University of Pisa, Via Bonanno Pisano 6, 56126 Pisa, PI, Italy; serenella.torre@phd.unipi.it (S.T.); lorenzo.flori@farm.unipi.it (L.F.); 2 Teregroup Srl, Via David Livingstone 37, 41122 Modena, MO, Italy; luca.usai.24@gmail.com (L.U.); gianni.lutzu@teregroup.net (G.A.L.); 3 Department of Biosystems and Agricultural Engineering, Robert M. Kerr Food and Agricultural Products Center, Oklahoma State University, 103 FAPC, Stillwater, OK 74078, USA; nazlim.aktay@okstate.edu (N.A.); nurhan.dunford@okstate.edu (N.T.D.)

**Keywords:** phycobiliproteins, C-phycocyanin, phycocyanobilin B, antioxidants, *Arthrospira* *platensis*, *Spirulina*, nutraceuticals

## Abstract

*Arthrospira platensis*, commonly known as *Spirulina*, is a photosynthetic filamentous cyanobacterium (blue–green microalga) that has been utilized as a food source since ancient times. More recently, it has gained significant popularity as a dietary supplement due to its rich content of micro- and macro-nutrients. Of particular interest is a water soluble phycobiliprotein derived from *Spirulina* known as phycocyanin C (C-PC), which stands out as the most abundant protein in this cyanobacterium. C-PC is a fluorescent protein, with its chromophore represented by the tetrapyrrole molecule phycocyanobilin B (PCB-B). While C-PC is commonly employed in food for its coloring properties, it also serves as the molecular basis for numerous nutraceutical features associated with *Spirulina*. Indeed, the comprehensive C-PC, and to some extent, the isolated PCB-B, has been linked to various health-promoting effects. These benefits encompass conditions triggered by oxidative stress, inflammation, and other pathological conditions. The present review focuses on the bio-pharmacological properties of these molecules, positioning them as promising agents for potential new applications in the expanding nutraceutical market.

## 1. Introduction

*Arthrospira* is a genus of multicellular, filamentous, and photosynthetic cyanobacterium with a helical or spiral shape. *Arthrospira platensis*, one of the most cultivated species, is commonly referred to as “*Spirulina*” [1].

*Spirulina* is a natural source of nutrients of commercial and industrial interest, belonging to both macro- and micro-nutrients. Particularly interesting in the nutraceutical field is the high content of *Spirulina* in proteins (greater than those in many animal-based foods) and the level of polyunsaturated fatty acids (PUFAs), polysaccharides, vitamins, minerals, and other health-promoting compounds such as polyphenols, carotenoids, and chlorophylls. Currently, due to this content of high-value molecules, the *Spirulina* biomass, after being recognized as safe for human consumption by the FDA, is largely used for realizing nutritional supplements and enriched food and cosmetics [2].

In addition to the commercial use of the *Spirulina* biomass, single *Spirulina* compounds or a class of compounds may be of interest for the nutraceutical market.

**Figure 1:**
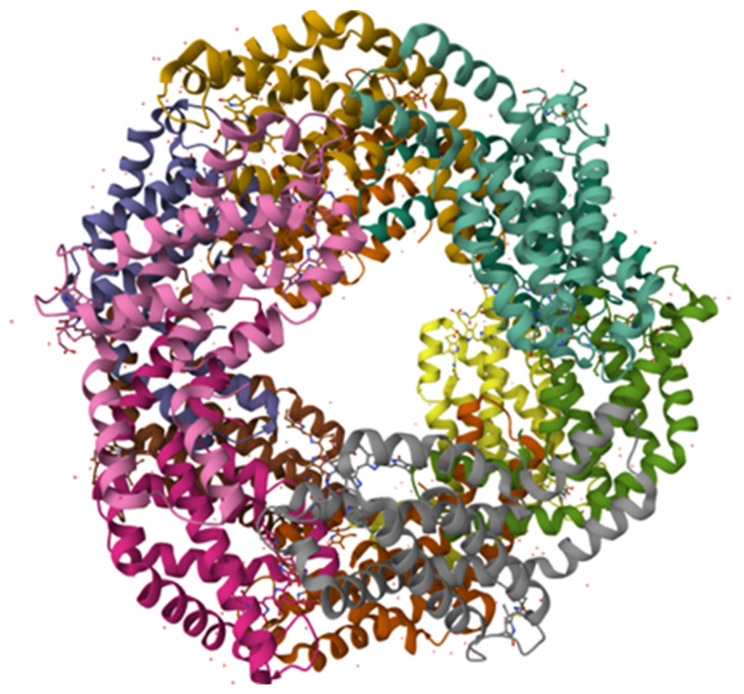
Crystal structure of C-PC from *Spirulina*. Reproduced from PDB database (https://doi.org/10.2210/pdb1gh0/pdb, accessed on 9 January 2024).

This is the case for the blue pigment C-phycocyanin (C-PC) (Figure 1).

It is a water-soluble, non-toxic, and vivid blue photosynthetic pigment employed in many products. It belongs to the phycobiliprotein (PBP) family and contains, as a chromophore, phycocyanobilin B (PCB-B).

The chromophores of PBPs share a chemical resemblance to bile pigments, which is why they are called phycobilins. These chromophores are open-chain tetrapyrroles linked to cysteine residues through thioester bonds [3].

Depending on its purity grade, C-PC finds applications in the food, cosmetic, pharmaceutical, and textile industries [4,5]. Various biological functions of C-PC have been described, and there are many potential uses of C-PC-rich extracts or purified C-PC for health benefits, which will be explored in the present review [6].

Currently, driven by the increasing demand for natural compounds, large-scale *Spirulina* cultivation is increasingly geared towards the production of C-PC. It is noteworthy that once C-PC has been extracted, the *Spirulina* biomass still retains substantial quantities of antioxidants, vitamins, and minerals; hence, the residual biomass can be a valuable natural ingredient in the formulation of nutraceutical and cosmeceutical products. These aspects make the C-PC market very attractive. To date, it is expected that the global C-PC market will grow to reach USD 245.5 million by 2027 and USD 279.6 million by 2030 [7].

**Figure 2:**
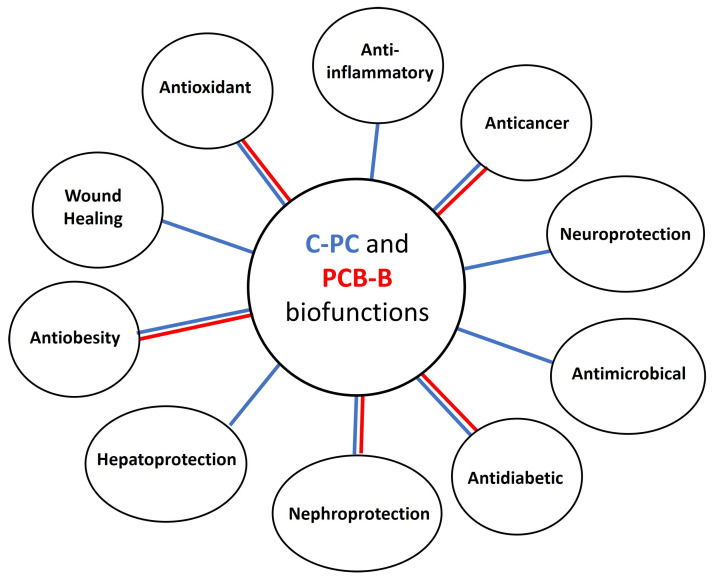
Schematic representation of potential therapeutic applications of C-PC and PCB-B (blue lines are in reference to C-PC while red lines to PCB-B).

C-PC and its chromophore PCB-B have been reported to exert active biological effects in different districts and in different pathological conditions [8]. The paragraphs below highlight the potential therapeutic applications of C-PC and PCB-B, as schematically represented in Figure 2.

All this considered, this review aims to present the latest advancements in understanding the role of *Spirulina* C-PC and PCB-B molecules within the growing nutraceutical market, focusing particularly on their primary effects on health. To achieve this goal, the most recent studies investigating the health-promoting properties of C-PC and PCB-B across various tissues/organs and under different pathological conditions will be critically evaluated.

An exhaustive overview of the effects of C-PC and PCB-B on different in vitro and in vivo models of human pathologies is provided in Table S1 (Supplementary Materials).

## 2. Health Promoting Effects

### 2.1. Antioxidant Effects by Spirulina-Derived C-PC or PCB-B

Many studies have recognized the potent antioxidant activity of *Spirulina*-derived C-PC and its chromophore PCB-B. Nevertheless, so far, studies have been carried out only on *in vitro* and *in vivo* models, and there are no specific clinical trials reported in the literature or in the *clinical trials.gov* database, neither with the whole C-PC nor with PCB-B.

Although the exact mechanism of their antioxidant action is not fully understood, most investigations suggest that C-PC has the capacity to scavenge free radicals, which are well known for causing oxidative damage to the body’s cellular system. Indeed, free radicals destabilize cells, causing DNA damage, cell death, as well as several linked disorders and diseases, since they are extremely reactive with several biological components [9]. The two main types of substances that lead to cellular oxidative damage are reactive nitrogen species (RNS) and reactive oxygen species (ROS), which can be developed as metabolic byproducts or are introduced or stimulated by exogenous factors like UV radiation [10].

The pioneering study by Bath and Madyastha reported that C-PC, with greater efficacy, and its chromophore PCB-B, to a lesser extent, scavenge the potent and inorganic toxin peroxynitrite [11]. On the contrary, Hirata et al. [12] attributed the antioxidant activity of C-PC to the tetrapyrrole PCB-B, based on their experimental results, showing that the intact protein and heat-denatured protein exerted the same antioxidant activity measured as the TROLOX equivalent. Moreover, in the same study, PCB-B was shown to be more effective in suppressing oxidation than other natural antioxidants, i.e., alfa–tocopherol, caffeic acid, and zeaxanthin [12].

Another study [13], using the ORAC (oxygen radical absorbance capacity) method, revealed the antioxidant activity of PCB-B to be greater than that of C-PC.

Moreover, in the study by Gabr et al. [14], the authors found the following order of antioxidant action: C-PC > PCB-B > phycocyanopeptide.

As regards the mechanisms involved in the antioxidant activity, PCB-B was reported to increase the expression of antioxidant enzymes and of the well-known antioxidant glutathione. In addition, experiments carried out both in vivo with rodents fed 100 mg of PCB-B [15] and in vitro on various cell culture models [16] showed that PCB-B is a potent inhibitor of NADPH oxidase, a producer of ROS.

Furthermore, Li [17] emphasized the antioxidant property of PCB-B through the inhibition of NOX, an activation of the heme oxygenase-1 (Hmox1), and the heme catabolic pathway for the production of the antioxidant bilirubin.

In evaluating the antioxidant activity of C-PC, Belokobylsky et al. suggested that this may be also related to the higher content of Se, an antioxidant on its own that is normally contained in the powder of *Spirulina*, and after binding PBPs, it may amplify their antioxidant capacity [18].

In other studies, C-PC demonstrated potent radical scavenging abilities against alkoxyl, superoxide, and hydrogen peroxide radicals and effectively inhibited lipid peroxidation [19,20,21].

In addition to the scavenging properties, another mechanism underlying the antioxidant activity of C-PC is probably due to its metal ion chelation capacity. Metals, especially transitional elements like iron, are involved in ROS production, working as catalysts in their synthesis [22]. As suggested by Chen et al. [23], the good metal chelating ability of C-PC is likely due to its particular aminoacidic composition, which sequesters metal ions, such as Fe^3+^. In this regard, Bermejo et al. [19], in examining the fluorescence spectra of C-PC in the presence and absence of iron, showed a massive decrease in the C-PC spectrum due to the metal, thus demonstrating the clear ability of PBP to chelate iron, which is a free radical promoter.

Since the crucial role of oxidative stress in the etiology of many disorders, C-PC and PCB-B were also evaluated for their protective activity against pathological oxidative damage. In the study of Bergandi et al., C-PC demonstrated good protection against inflammatory and oxidative stress at 1 μM on human bronchial and prostate epithelial cells, also revealing an enhanced activity when associated with palmitoylethanolamide [24].

Haemolysis, as a model of chemically induced oxidative damage on erythrocytes, was studied by Pleonsil et al. [25], who investigated the protective activities of both native C-PC and recombinant Apo-C-PC (beta subunit). Their results reveal that the native C-PC was a powerful inhibitor (almost totally) at 1 μM, and it was more protective than recombinant Apo-C-PC. This protection was suggested to be due to the presence of cysteine residues on the protein surface, counteracting oxidation via thiol groups on the molecule [26].

Another interesting protection by C-PC (10 mg mL^−1^) was observed by Puglisi et al. [27], who studied the damage caused by UVA radiation exposure on in vitro keratinocytes and skin fibroblasts through the measurement of ROS levels. This finding suggests a potential use of C-PC as an antioxidant in skincare products, reducing photo-aging.

All these activities reveal that C-PC may be a useful molecule for the treatment of a variety of diseases caused by oxidative stress. Some specific protective activities on other tissues/organs are reported in subsequent paragraphs of this review.

### 2.2. Anti-Inflammatory Effects by Spirulina-Derived C-PC or PCB-B

Many results report anti-inflammatory activity, particularly deriving from different animal models, while no clinical studies have evaluated the preventive effect of C-PC or PCB-B against inflammatory-based diseases. The anti-inflammatory activity of C-PC was first recognized in the study by Romay et al. [20], who observed the inhibition of paw inflammation and edema induced by glucose-oxidase local injections when 100 and 200 mg kg^−1^ of C-PC was administered *per os* or i.p to Oncins France 1 (OF_1_) mice. Moreover, many other studies have confirmed the anti-inflammatory property of C-PC in different models.

In a study performed on a carrageenan-induced thermal hyperalgesia model, Shih and colleagues reported that C-PC (30 or 50 mg kg^−1^, i.p.) had an anti-hyperalgesic activity closely related to its anti-inflammatory profile. In particular, C-PC inhibited the inducible over-expression of nitric oxide synthase (iNOS) and cycloxygenase-2 (COX-2) after 4 h from the pro-inflammatory stimulus, while reducing the formation of TNF-α, prostaglandin E2, and nitrate and decreasing myeloperoxidase activity in male Sprague Dawley rats [28].

A protective effect of C-PC, as well as of the aqueous extract from *Spirulina*, was also demonstrated against an inflammatory condition in a lipopolysaccharide (LPS)-induced activation of BV-2 microglial cells [29]. This study showed that the expression of iNOS and COX-2 was inhibited and TNF-α and IL-6 were down-regulated in BV-2 microglial cells treated with C-PC (85, 125, and 150 μg mL^−1^ for 24h). The latter finding suggests a possible application of C-PC towards neurodegenerative pathologies, such as Alzheimer’s disease (AD) and Parkinson’s disease (PD), where a microglial neuro-inflammatory component has been recognized.

The modulation of inflammatory processes regulated by COX-2, NF-kB, and IL-6 was also demonstrated for the neuroprotective activity of C-PC in a tributyltin chloride-induced neurotoxicity model involving male Wistar rats [30].

Furthermore, C-PC (150 mg kg^−1^) daily intake ameliorated dextran sulfate sodium (DSS)-induced colitis and relieved associated bloody diarrhea and weight loss in male C57BL/6 mice [31]. The partial resolution of the pathological scenario was accompanied by the reduction in inflammatory cytokines (IL-6, TNF-α, MCP-1, and IL-10) and the suppression of NF-κB nuclear translocations.

Similar mechanisms were observed in an acute lung injury in male C57BL/6 mice, where C-PC (100, 200, and 400 mg kg^−1^, i.p.) reduced the activation of the NF-κB/NLRP3 pathway and the formation of the NLRP3 inflammasome complex [32]. The levels of IL-6, TNF-α, and other inflammatory and oxidative markers were also found to be decreased in a model of acute pulmonary fibrosis in rats and in the same mouse strain indicated above [33].

In another study, C-PC was able to contain neuro-inflammation and upregulate levels of the brain-derived neurotrophic factor (BDNF) and insulin-like growth factor-1 (IGF-1) after the administration of streptozocin (STZ) in female Wistar rats [34].

Furthermore, two different investigations demonstrated that C-PC also exerted anti-inflammatory effects through the inhibition of the arachidonic cascade: one showed the inhibitor activity of the apoprotein C-PC on COX-2 via in vitro assays; the second one reported a reduced formation of leukotriene B4 via LOX in an in vivo mouse ear inflammation test [35,36].

Further experimental evidence also suggests a direct modulation by C-PC on TLR2 and TLR4, which regulate the secretion of pro-inflammatory cytokines through p38, NF-κB, and ERK-AP-1 transcription factors [37]. Nevertheless, another study suggests that C-PC administered *per os* at a dosage of 50 mg kg^−1^ in male C57BL/6 mice may inhibit the NF-κB pathway by a direct action on this factor [33].

C-PC is also able to modulate expressions of the BCL-2 family of proteins, inhibiting caspase-3 and reducing inflammation-induced tissue apoptosis both in in vitro and in vivo models [38,39].

With respect to PCB-B, when evaluated at 0.1–1 mg kg^−1^ i.p., the chromophore exerted anti-inflammatory activity in a mouse model of autoimmune encephalomyelitis (EAE), demonstrating a protective effect on myelin [40] and suggesting that PCB-B, in combination with other drugs, could be used in the treatment of MS. Furthermore, in a model of acute cerebral hypoperfusion conducted on male Wistar rats, PCB-B decreased the levels of pro-inflammatory genes when administered at 47 or 213 μg kg^−1^ i.p. for 30 min and 1, 3, and 6 h after the permanent occlusion of the bilateral common carotid arteries [41]. Overall, the results obtained in this study suggest that the potential of PCB-B as an acute disease-modifying drug against ischemic strokes should be further investigated.

### 2.3. Anticancer Effects by Spirulina-Derived C-PC or PCB-B

The anticancer activity of C-PC has been largely described and molecular mechanisms investigated, but until now, there has been no investigation on human subjects. Only one study described the activity for the whole *Spirulina*, which looked specifically at the effects of the microalga on oral carcinogenesis, in particular, leukoplakia [42]. The chemopreventive activity of Spirulina (1 g/day for 12 month) in reversing oral leukoplakia in pan tobacco chewers in Kerala, India was evaluated. A complete regression of lesions was observed in 20 of 44 (45%) evaluable subjects supplemented with SF, as opposed to 3 of 43 (7%) in the placebo arm [42].

The ability of C-PC (0.2 μg mL^−1^) to induce an anticancer effect in the hepatocarcinoma cells HepG2 appeared to be due to its capacity to affect the mitochondrion pathways promoting necrosis and apoptosis [43]. In MDA-MB-231 breast cancer cells, 100, 200, and 400 µg mL^−1^ of C-PC significantly reduced actin filament formation and cell migration [44]. Moreover, C-PC may act as an inhibitor of COX-2, which plays a key role in some tumor progressions and chemical resistances related to metastasis and angiogenesis, as demonstrated in rat xenograft models, after the oral administration of 200 mg kg^−1^ of C-PC [45].

C-PC also showed the ability to down-regulate matrix metalloproteases (MMP-2 and MMP-9), which are necessary for the invasion into surrounding tissues and tumor metastasis. Furthermore, the pigment inhibits metastasis formation by binding cadherin-E a relevant molecule in the migration process, as observed in colon cancer and melanoma [46]. In this latter study, the authors also showed that 100, 200, and 400 µg mL^−1^ of C-PC also interact with the BRAF and MEK pathways and inhibit cyclin-dependent kinase (CDK) 4 and 6 in the murine cell line B16F10. The ability of C-PC to affect cell cycle progression was also reported in other cancer cell lines, i.e., colorectal tumor cells HT-29, lung cancer cells A549, and MDA-MB-231, leading to a G0/G1 phase arrest by reducing the expression of the CDK inhibitor 1 (p21) as well as by down-regulating cyclin E and CDK2 expression in a concentration range of 40–80 μg L^−1^. In addition, C-PC induced G2/M cell cycle arrest in the human ovarian cancer cell SKOV-3 and in the pancreatic cancer cell PANC-1 and enhanced the efficacy of chemotherapeutic agents in HepG2, LNCaP, A549, and HeLa cells [47,48,49,50,51].

Moreover, a recent study analyzed the effects of 0.0625–2 mg mL^−1^ of C-PC and its chromophore alone (3.125–100 μg mL^−1^ of PCB-B) on human breast cancer cells (MCF-7). The authors observed a similar decrease in cell viability and, in both cases, an increased expression of apoptotic genes [52]. So, this evidence suggests a role of PCB-B in the anticancer effects of C-PC.

Finally, a very recent study showed that PCB-B may act as a photosensitizer, and its anticancer effects are greater in vivo than *in vitro*, which is probably due to the additional in vivo anti-inflammatory and immune-modulating activities of the molecule [53,54].

### 2.4. Hypolipidemic, Anti-Obesity, and Anti-Diabetic Effects of Spirulina-Derived C-PC or PCB-B

Several studies on human subjects have highlighted the correlation between the intake of *Spirulina* or its protein extracts and a significant decrease in all undesirable lipid fractions such as total cholesterol, triglycerides, LDL-C, and other non-HDL-Cs as well as in the body weight of obese individuals [55,56,57,58]. Moreover, a meta-analysis demonstrated that *Spirulina* has normo-glycaemic and therapeutic effects against metabolic syndrome, improving fasting glucose and insulin levels and also improving total cholesterol, LDL-C, VLDL-C, and HDL-C [59]. Although no evidence regarding the effects of C-PC on human metabolism and metabolic disorders has yet been obtained through clinical studies, many reports highlight these effects on different models.

In 2005, Nagaoka and collaborators [60] were the first authors to suggest C-PC as the protein in *Spirulina* that is responsible for the down-regulatory effects of cholesterol. In a human epithelial cell line “Caco-2” model, these authors observed that a concentrated *Spirulina* extract (SPC) was able to significantly reduce the absorption of bile acids and cholesterol by intestinal cells. The results obtained *in vitro* were confirmed in vivo in the same study, showing that a group of rats that were fed SPC had a higher HDL blood concentration and significantly lower total lipids and cholesterol concentrations in the liver without showing any differences in body and liver weights when compared with the group fed casein instead of SPC.

Zhao et al. [61], through an investigation involving mice fed a high fat diet, reported that a *Spirulina* protein extract induced an evident anti-obesity effect and a different expression in the brain and liver for a number of genes coding for proteins involved in the metabolic pathway of the gut–brain–liver axis. In particular, the most significant change in the brain was a decrease in the gene coding for the acyl-CoA dehydrogenase medium chain (ACADM), while in the liver, the most relevant changes were increases in the genes coding for resistin (Retn), the fatty acid binding protein 4 (Fabp4), the peroxisome proliferator-activated receptor delta (PPAR-delta or Ppard), and the solute carrier family 27 member 1 (Slc27a1).

Recently, further in vivo studies were carried out using C-PC or its chromophore PCB-B from *Spirulina*. In particular, an Egyptian study on diabetic rats orally administering C-PC (50 mg kg^−1^) or PCB-B (982 µg Kg^−1^) showed a significant reduction in total cholesterol and triglycerides, insulin resistance (evaluated with the Homeostatic Model Assessment-HOMA- score), and fasting blood glucose in both groups, demonstrating that C-PC and isolated PCB-B possess hypolipidemic and hypoglycemic activities [62].

It has also been shown that the dietary treatment of obese rats with C-PC decreases serum total cholesterol, fasting glucose, and body weight, hypothesizing the benefits of C-PC on insulin resistance and hepatic steatosis [63].

A recent study in mice that were fed a high fat diet and that underwent, after 16 weeks, a significant increase in body weight gain compared to a control (mice fed with normolipid diet), intragastric C-PC (500 mg kg^−1^ daily) was able to protect against the body weight gain, serum leptin, and the retroperitoneal fat depot, also reducing the levels of the serum NF-kB p-50 subunit, according to an anti-inflammatory activity [64].

With regards to, more specifically, investigations on the protective effects on diabetes and its complications, Zheng et al. [65], in a model of diabetic nephropathy, demonstrated protection not only by C-PC but also by PCB-B. The oral administration of 300 mg kg^−1^ of C-PC to mice for 10 weeks played a protective role against albuminuria and renal mesangial expansion, also normalizing the expression levels of transforming growth factor beta (TGF-β) and fibronectin by suppressing oxidative stress. The results also reveal a specific role for the chromophore PCB-B (orally administered at 15 mg kg^−1^ for two weeks), which was shown to inhibit the activity of NOX, the major cause of cellular ROS formation, which is linked to vascular complications in diabetic subjects, and Nox4, which is responsible for renal dysfunction [65].

The antidiabetic effects of C-PC were also demonstrated by Ou et al. [66], who orally administering KK-Ay mice 100 mg kg^−1^ of the protein extracted from *Spirulina* once daily for three weeks. The mouse strain KK-Ay is a model for the mutation of the “agouti” protein, an inverse agonist of melanocortin receptors, which confers a disease phenotype that resembles obesity and diabetes in humans. By feeding these mice C-PC, improvements in many parameters including body weight, fasting glucose levels, and glycosylated protein levels 24 h after treatment were obtained [66]. All results were explained by the effect of C-PC on glucokinase (GK), an enzyme present in two different isoforms in the liver and pancreas, whose transcription is activated differently but is influenced by the presence of insulin and glucagon.

In subsequent studies, Ou et al. [67,68] observed that the treatment of alloxan-induced diabetic mice with 100 mg kg^−1^ of C-PC enhanced the GK and glucokinase regulatory protein (GKRP) levels in the liver and pancreas. These data suggest that C-PC may activate, in diabetic mice, the insulin signaling pathway and GK expression in the pancreas and liver.

A protective effect of C-PC was also induced, *in vitro*, by the activation of the nuclear erythroid-related factor (Nrf2), leading to an increased expression of the antioxidant enzymes heme-oxygenase (HO) and glyoxalase (Glo). These enzymes control oxidative stress and inflammation, two conditions that favor the onset of diabetes [69].

An insulin-sensitizing effect of C-PC, modulating the PI3K pathway, was suggested in a study investigating the methylglyoxal (MG)-induced cell dysfunction on rat insulinoma beta-cells INS-1 [70].

Thereafter, the positive effects of C-PC in the treatment of diabetes were confirmed by a further study conducted on both cellular and animal models [71]. It was observed that specifically in mice with type 2 diabetes mellitus (T2DM) that were treated orally with 200 mg kg^−1^ and in insulin-resistant cells, C-PC increased insulin sensitivity and the cellular use of glucose through the activation of AMP-activated protein kinase (AMPK), which, once active, promotes autophagy, and of serine/threonine kinase 1 (AKT), which is part of the phosphatidylinositol-3 kinase (PI3K) pathway and is relevant in the regulation of the glyco-metabolism [71].

Using another in vivo model, for streptozotocin (STZ)-induced diabetic Wistar rats, Husain et al. [72] evaluated the ability of C-PC to prevent diabetes and inhibit glycation. High concentrations of glycated hemoglobin (HbA1c) in STZ-induced diabetic rats decreased after a daily treatment of 100–200 mg kg^−1^ of C-PC. The total cholesterol, triglycerides, LDL-C, glutamic-oxaloacetic transaminase (SGOT), alkaline phosphatase (ALP), aspartate aminotransferase (SGPT), and total bilirubin levels were also reduced, suggesting a hepatoprotective function by C-PC as the possible preventive basis against diabetes complications [72].

In addition to the reported targets above, an inhibition by PBPs of the enzymatic activity of dipeptyl-peptidase (DPP)-IV was recently reported, from *in vitro* studies, in two papers. The inhibition of DPP-IV, which is an enzyme involved in the incretins (metabolic hormones that stimulate a decrease in blood glucose levels) metabolism, is currently one of the innovative strategies to control the development of diabetes. The inhibitory effects of C-PC and allophycocyanin (APC) hydrolysates were observed on human enterocyte cells [73,74].

In a recent review, Ziyaei and collaborators [75], in examining the molecular mechanisms involved in the anti-diabetic effects described for C-PC, suggested that a reduction in β-cell apoptosis, an improvement of their function, and a regulation of the hepatic glucose metabolism were the main mechanisms involved in C-PC activity.

### 2.5. Hepatoprotection by Spirulina-Derived C-PC or PCB-B

As for the other effects that have not yet been described, there are no clinical trials on the hepatoprotective effects of C-PC. Nevertheless, several pieces of experimental evidence, both from *in vitro* and in vivo models, seem to highlight an interesting hepatoprotective effect of the *Spirulina*-derived PBP.

The first evidence was reported by Vadiraja and coworkers in 1998 [76]. These authors observed that a single dose of C-PC administered intraperitoneally (200 mg kg^−1^) significantly reduced CCl_4_ and R-(1) pulegone-induced hepatotoxicity in rats by providing protection to liver enzymes. Accordingly, C-PC alleviated a CCl_4_-induced liver injury in the human hepatocyte cell line L02 and in vivo mice and rats by protecting liver cells against free radical damage, oxidative stress, and inflammatory infiltration [77,78]. Similarly, the intraperitoneal pre-administration of C-PC (50–200 mg kg^−1^) significantly reduced necrosis and inflammation during intraperitoneal galactosamine-induced hepatitis in female Wistar rats [79].

Moreover, C-PC significantly reduced Kupffer cell phagocytosis and oxidative stress following colloidal carbon uptake in isolated and perfused mouse liver models [80] and protected against gibberellic acid (a dangerous pesticide)-induced hepatic damage, improving the antioxidant defense system, liver enzymes, and hepatocyte structure [81]. In another study, the oral administration of C-PC (50 mg kg^−1^ of the body weight, twice in 24 h intervals) to rats with thioacetamide-induced hepatic encephalopathy increased antioxidant defense responses and ameliorated encephalopathy [82].

Despite the evidence for the potential for liver protection by C-PC, an effect of the chromophore component PCB-B against liver fibrosis has only been hypothesized. Based on the PCB-B metabolism, which produces biliverdin/bilirubin homologues modulating NADPH oxidase [83], PCB-B was speculated to inhibit liver fibrosis, probably decreasing stellate cell proliferation and transformation into collagen-secreting myofibroblasts [84].

### 2.6. Neuroprotective Activity by Spirulina-Derived C-PC or PCB-B

Currently, one clinical trial (PROPERTY) evaluating the efficacy of C-PC preparation (Phycocare^®^) against the neurotoxicity of oxaliplatin-based chemotherapy in patients with gastrointestinal cancer is ongoing (clinicaltrials.gov ID NCT05025826). On the contrary, no clinical studies with PCB-B are reported. On the other hand, many pieces of evidence on the neuroprotective potential of C-PC or its chromophore PCB-B derive from non-human studies. C-PC was reported to reduce neuroinflammation in a rat model of focal cerebral ischemia/reperfusion (I/R) and acute brain hypoperfusion [85]. In particular, C-PC was i.p. administered at 50, 100, and 200 μg kg^−1^ for up to 6 h post-stroke. C-PC significantly reduced the infarct volume and neurological deficit in a dose-dependent manner and improved the exploratory activity of I/R rats. Furthermore, C-PC down-regulated the expression of pro-inflammatory genes (IFN-γ, IL-6, IL-17A, CD74, and CCL12) and upregulated immune-suppressive genes (Foxp3, IL-4, and TGF-β) in hypoperfused brain areas [85].

Similar results were obtained by Pavón-Fuentes et al., who tested PCB-B that protected PC12 neuronal cells against oxygen and glucose deprivation. In Wistar rats, PCB-B at 50, 100, and 200 μg kg^−1^ administered i.p. (30 min and 1, 3, and 6 h after the I/R ischemic event) significantly decreased the brain infarct volume, limited exploratory behavior impairment, and preserved viable cortical neurons in ischaemic rats in a dose-dependent manner compared to a vehicle group [86].

The supplementation of C-PC (180 mg kg^−1^ day^−1^ for 28 days through the diet) to rats with a partial injury induced at the level of T12 improved the ultrastructure of the spinal cord gray matter when compared to the control group, indicating the neuroprotective potential of C-PC in reducing the effects of spinal cord injury and causing functional recovery. The group, supplemented with C-PC, thus revealed a better improvement in the fine ultrastructure of the spinal cord gray matter when compared to the control group, thereby suggesting the neuroprotective potential of *C-PC* in mitigating the effects of spinal cord injury and inducing functional recovery [87].

In a 1-methyl-4-phenyl-1,2,3,6-tetrahydropyridine (MPTP)-induced zebrafish PD model, C-PC significantly counteracted the loss of dopamine neurons and cerebral vessels, reducing the locomotor impairment in PD zebrafish [88].

Another study demonstrated that a protein-enriched fraction (SPF) of *Spirulina*, at 5 and 10 mg kg^−1^ administered for 15 days *per os*, reduced brain oxidative stress, increasing the striatal expression of tyrosine hydroxylase and the dopamine transporter, while reducing hippocampal inducible NOS, COX-2, and glial fibrillary acidic protein expressions. So, these data also suggest that C-PC, through its brain anti-inflammatory and antioxidant actions, exerts neuroprotective effects that could benefit patients affected by neurodegenerative diseases, like PD [89].

The neuroprotection of C-PC was also assessed in MS models. In identifying novel oral disease-modifying therapies for MS, targeting both its inflammatory and neurodegenerative components is still a major goal. A total of 200 mg kg^−1^ of C-PC administered orally once a day for 7 consecutive days before the ischemic event to Lewis rats with experimental autoimmune encephalomyelitis (EAE) significantly improved clinical signs and restored the motor function of the animals. Furthermore, C-PC positively modulated oxidative stress markers measured in a brain homogenate and serum and protected the integrity of cerebral myelin sheaths [90].

A similar protective effect was demonstrated in a murine model of EAE [91]. After the daily administration of C-PC at 2, 4, or 8 mg kg^−1^ i.p, the clinical deterioration of animals was limited, reducing the inflammatory infiltrates of the spinal cord tissue, down-regulating the IL-17 expression in the brain tissue and serum, and positively modulating the expression of genes related to remyelination [91].

Hence, supplemental C-PC may have considerable potential for preventing or slowing the progression of a range of neurodegenerative disorders.

### 2.7. Other Pharmacological Effects by Spirulina-Derived C-PC or PCB-B

#### 2.7.1. Effects on Eyes

A cataract is a cloudy area in the eye lens inducing a decrease in vision and classically is associated with ageing and eye exposure to UV rays. C-PC (100–200 mg kg^−1^ i.p.) from *Spirulina* showed a protective potential against a model of cataractogenesis (selenite-induced in rats) by maintaining reduced glutathione (GSH) levels and antioxidant enzymatic activity and inhibiting lipid peroxidation [92]. Subsequent results confirm that C-PC (200 mg kg^−1^ i.p.) was able to prevent cataractogenesis triggered by sodium selenite, maintaining lens transparency through the regulation of the apoptotic cascade and the expression of the mRNA of redox genes [93].

#### 2.7.2. Effects on Ears

Tinnitus, the perception of noise in the absence of acoustic stimulation, is an ear disorder that can become disabling for those who suffer from this ailment. A particular type of tinnitus may be induced by drugs such as salycilate. Salicylate-induced tinnitus appears to be, in part, associated with an increased K^+^-Cl^−^ co-transporter 2 (KCC2) mRNA expression in cochlear areas. Results obtained on animal models suggest that a dietary supplementation with C-PC (130 mg kg^−1^ *per os*) is able to reduce salicylate-induced tinnitus and reduce the mRNA expression of the N-methyl D-aspartate (NMDA) receptor subtype 2B (NR2B), TNF-α, IL-1β, and COX-2 in the cochlea and the inferior colliculus of mice [94].

Another protective activity of C-PC on the auditory system was observed in a recent study where C-PC *in vitro* (0.1–20 µg mL^−1^) protected HEI-OC1 cochlear cells from cisplatin-induced ototoxicity, inhibiting apoptotic processes, preventing ROS production, and promoting mitochondrial functionality [95].

#### 2.7.3. Effects on Fertility

In recent studies, C-PC has highlighted a positive impact on the processes underlying both female and male infertility. In particular, C-PC, through an antioxidant activity, partially improved the reduced female fertility induced in a mouse model by using D-galactose, an ageing inducer. In this model, C-PC (250 and 500 mg kg^−1^ day^−1^ *per os* and i.p.) reduced the MDA content, improved mitochondrial distribution, and reduced apoptotic mechanisms through ROS containment in oocytes [96]. Similarly, in obese female mice, C-PC (500 mg kg^−1^ day^−1^ *per os*) improved the reproductive ability, restoring ovary/oocyte quality by an enhanced level of antioxidant enzymes and reduced levels of ROS [97].

For the male reproductive system, *in vitro* evidence using a spermatogonia germ cell line (GC-1 spg) demonstrated that C-PC, especially its β-subunit, plays a protective role against H_2_O_2_-induced cell damage. Indeed, C-PC (0.25–1 mg mL^−1^) prevented ROS overproduction and mitochondrial potential loss and reduced cell necrosis [98].

#### 2.7.4. Effects on Kidneys

A prophylactic role of C-PC (100 mg kg^−1^ i.p.) against oxalate-induced in vivo renal calculi formation and injury and *in vitro* (C-PC 5–50 mM) kidney epithelial cell damage, through an antioxidant activity, was reported by Farroq and collaborators [99,100]. C-PC (5–50 mg kg^−1^ i.p.) was also able to decrease the toxicity in kidneys induced by the anticancer drug cisplatin, as revealed by several in vivo studies [101,102,103]. Also in these cases, the responsible mechanism is reported to be the inhibition of oxidative stress, with protection against mitochondrial dysfunction.

Recently, in the study by Oumayma et al. [104], C-PC, administered at 25 and 50 mg kg^−1^ to Wistar rats, exerted a protective action in kidneys (in addition to a positive effect on the liver and behavior) against the oxidative stress-linked toxicity by ethanol.

An effect similar to that of C-PC was exerted by PCB-B against diabetic nephropathy, as already reported in the “Anti diabetes” paragraph of this review [65]. Moreover, PCB-B (0.75–3 mg kg^−1^ *per os*) was shown to be responsible for the nephroprotective action of the whole C-PC on acute kidney injury caused by mercury [105].

#### 2.7.5. Effects on Endothelium

C-PC (a 2500–10,000 mg kg^−1^ diet for 25 weeks) was able to prevent hypertension in a rat model of metabolic syndrome by enhancing the eNOS expression in aorta [106]. Moreover, the antioxidant action of C-PC (100 mg kg^−1^ *per os*) was able to reduce the damage on rat endothelium induced by a chronic kidney disease, promoting an anti-hypertensive effect [107].

#### 2.7.6. Effects on the Skin

C-PC regulated the transcription of the urokinase-type plasminogen activator (uPA), which is relevant in the process of wound healing [108]. Madhyastha et al. [109] demonstrated that C-PC (75 µg mL^−1^) was able to increase fibroblast proliferation and migration, revealing an accelerated wound healing in an in vivo mouse model.

C-PC also demonstrated a photoprotective ability, particularly a protection against UV ray-induced damage *in vitro* on keratinocytes [27,110] and on in vivo UVB-exposed mice [111].

#### 2.7.7. Antimicrobial Effects

C-PC has been evaluated in different studies for its potential antimicrobial *in vitro* effects. Activities against bacteria involved in acne and in dermatitis [112] and against bacteria with a high level of intrinsic resistance to most antibiotics, such as *Pseudomonas aeruginosa, Klebsiella pneumoniae*, and others, were reported [113,114].

Interestingly, a recent paper also suggested a concentration-dependent *in vitro* antimicrobial activity by C-PC (from 7.8 to 1000 μg mL^−1^) against a microorganism involved in gingival infections (*Porphyromonas gingivalis*). In this study, C-PC was associated with a photodynamic therapy with laser irradiation, and C-PC showed antimicrobial activity *per se* and a photosensitizing activity, revealing its possible use in infective prevention after implants or other oral conditions [115].

In addition to the antibacterial effects, an *in vitro* anti-HIV activity by C-PC on a HeLa-modified cell line (TZM-bl) was reported [116]. Interestingly, C-PC was also used for the green synthesis of silver nanoparticles with antibacterial and anti-mycotic activities [117].

## 3. Conclusions

*Spirulina* is currently available in the nutraceutical market, presenting itself in various forms such as a dry powder, capsules, and tablets. The health-promoting effects of *Spirulina* are often attributed to the presence of C-PC, the primary PBP in this cyanobacterium.

C-PC is easily extractable in water and is currently marketed mainly as a natural and safe food colorant. Nevertheless, numerous studies reported in the literature suggest potential applications of C-PC in preventing or even treating various diseases. The primary activities associated with C-PC hinge on its remarkable ability to protect cells from oxidative damage and inflammatory conditions. Consequently, C-PC may exert protective effects on human health, preventing and counteracting conditions such as neurodegenerative disorders, diabetes, obesity, hypertension, hyperlipidemia, and hepatic or renal failure. In addition, C-PC administration has shown promise in addressing ageing or exposure to toxic substances and other disorders including cataracts, hearing loss, and infertility. Experiments exploring the digestion of C-PC and the use of the isolated chromophore PCB-B have intriguingly indicated a relevant role for the tetrapyrrole compound in the overall activities of C-PC. In the case of peroxynitrite scavenger activity, the effect of free PCB-B was even greater than that of C-PC. Moreover, PCB-B was shown to be responsible for the nephroprotective action of the whole C-PC. In addition, the anticancer activity of C-PC against human breast cancer cells was similar to that observed with isolated PCB-B, revealing, also in this case, PCB-B to be probably responsible for the activity by C-PC. This observation raises the possibility of utilizing the isolated chromophore as an ingredient for dietary supplementation or functional foods instead of C-PC. Unlike C-PC, which is a protein susceptible to digestion in the gastrointestinal tract and with limited bioavailability, free PCB-B offers potential advantages.

Moreover, there is evidence suggesting additional noteworthy effects of C-PC or its isolated chromophore PCB-B, such as its anticancer and anti-microbial properties. From a nutraceutical point of view, these activities could be crucial in considering these molecules as chemo-preventive agents.

Although many health effects exerted by C-PC and PCB-B are also reported for *Spirulina* used as a dried biomass, the evidence obtained with the isolated molecules or with enriched extracts may represent a new strategy in the use of the cyanobacterium by the nutraceutical industry. This could also open up the possibility of exploiting the same biomass of *Spirulina* for different markets. The aqueous extraction of C-PC, in fact, does not alter the other properties of *Spirulina*. For example, a previous C-PC extraction procedure leaves unchanged the possibility to use the *Spirulina* biomass for another extraction procedure for oil production.

Despite a wealth of literature describing the potential nutraceutical value of *Spirulina*-derived C-PC and PCB-B, only a limited number of studies currently clarify the pharmacokinetic profile of specific formulations and the optimal dosage in humans. Consequently, more clinical trials are urgently needed to facilitate the successful incorporation of these natural compounds as active ingredients in health-promoting products on the market.

## Supplementary Materials

The following supporting information can be downloaded at: https://www.mdpi.com/article/10.3390/nu16111752/s1, Table S1: Effects of C-PC or PCB-B on different disease models [20,24,25,26,27,28,29,30,31,32,33,34,36,38,39,40,41,43,44,45,46,47,48,49,50,51,52,62,63,64,65,66,67,68,71,72,76,77,78,79,80,81,82,85,86,87,88,90,91,92,93,94,95,96,97,98,99,100,101,102,103,104,105,106,107,108,109,111,112,113,114,115,116,117].

## Abbreviations

ACADM	Acyl-CoA dehydrogenase medium chain
APC	Allophycocyanin
AD	Alzheimer’s disease
AKT	Serine/threonine kinase 1 (also named protein kinase (PK)-B)
ALP	Alkaline phosphatase
AMPK	AMP-activated protein kinase
APC	Allophycocyanin
BRAF	B-Raf proto-oncogene, serine/threonine kinase
BDNF	Brain-derived neurotrophic factor
C-PC	Phycocyanin C
CDK	Cyclin-dependent kinase
COX-2	Cycloxygenase-2
DPP	Dipeptidyl-peptidase
DSS	Dextran sulfate sodium
eNOS	Endothelium nitric oxide synthase
EAE	Experimental autoimmune encephalomyelitis
Fabp	Fatty acid binding protein
FDA	Food and drug administration
GK	Glucokinase
GKRP	Glucokinase regulatory protein
Glo	Glyoxalase
GSH	Cerebellar glutathione
HbA1c	Glycated hemoglobin
HO	Heme-oxygenase
IGF	Insulin-like growth factor-1
iNOS	Inducible nitric oxide synthase
KCC2	Potassium-chloride transporter member 5
LPS	Lipopolysaccharide
MDA	Malondialdehyde
MEK	Mitogen-activated protein kinase kinase
MG	Methylglyoxal
MMP	Metalloproteases
MPTP	1-methyl-4-phenyl-1,2,3,6-tetrahydropyridine
NADPH	Nicotinamide adenine dinucleotide phosphate hydrogen
NMDA	N-methyl D-aspartate receptor
Nrf	Nuclear erythroid–related factor
NOX	NADPH oxidase
OF	Oncins France 1 mouse
ORAC	Oxygen radical absorbance capacity
PARP-1	Poly (ADP-ribose) polymerase-1
PBPs	Phycobiliproteins
PCB-B	Phycocyanobilin B
PD	Parkinson’s disease
PI3K	Phosphatidylinositol-3 kinase
PUFA	Polyunsaturated fatty acid
Ppard	Peroxisome proliferator-activated receptor delta
Retn	Resistin
RNS	Reactive nitrogen species
ROS	Reactive oxygen species
SGOT	Glutamic-oxaloacetic transaminase
SGPT	Aspartate aminotransferase
Slc	Solute carrier family
SOD	Superoxide dismutase
SPC	*Spirulina platensis* concentrate
SPF	Protein enriched fraction
STZ	Streptozotocin
T2DM	Type 2 diabetes mellitus
TGF-β	Transforming Growth Factor
UVA	Ultraviolet A
