# Supplementary material for: Nutraceutical Features of the Phycobiliprotein C-Phycocyanin: Evidence from Arthrospira platensis (Spirulina)
Source: Nutrients. 2024 Jun 3;16(11):1752. doi: 10.3390/nu16111752 (PMC11174898; doi:10.3390/nu16111752)
Supplement: Supplementary file 1 [file nutrients-16-01752-s001.zip › nutrients-3025401-supplementary.pdf]

Tab. S1: Effects of C-PC or PCB-B on different disease models

Effect	*In vitro* / *in vivo* model	Mechanism of action	Dose / concentration evaluated	Ref
Anti-oxidant, anti-inflammatory	OF_1 mice and Sprague Dawley rats (edema-induced by glucose oxidase)	Scavenging activity	C-PC 50 - 200 mg kg^{-1} (os or i.p.)	[20]
Anti-oxidant, anti-inflammatory	Human lung (Beas-2B) and prostate (HPrEpiC) epithelial cell lines (LPS-stimulated or polyinosinic:polycytidylic acid-stimulated)	Reduced COX-2 activity and ROS production, increased glutathione levels	C-PC 1 μM	[24]
Anti-oxidant	Isolated erythrocytes (haemolysis)	Cysteine-mediated scavenging activity	C-PC 1 μM	[25,26]
Anti-oxidant	Human fibroblasts and keratinocytes (UVA exposure skin photo-aging)	Anti-ROS activity	C-PC 10 mg mL^{-1} fractionated PC 0.08 and 0.30 mg mL^{-1}	[27]
Anti-hyperalgesic and anti-inflammatory	Sprague-Dawley rats (carrageenan-induced thermal hyperalgesia)	Inhibition of iNOS and COX-2; reduction of TNF-α, prostaglandin E_2, nitrate and myeloperoxidase activity	C-PC 30 or 50 mg kg^{-1} (i.p.)	[28]
Anti-inflammatory	BV-2 microglial cell line (LPS-activated inflammation)	Inhibition of iNOS and COX-2 expression; downregulation of TNF-α and IL-6	C-PC 85 -150 μg mL^{-1} for 24h	[29]
Anti-oxidant, anti-inflammatory	Wistar rats (tributyltin chloride-induced neurotoxicity)	Down regulation of COX-2, NF-kB, IL-6 and Nrf2	C-PC 50 mg kg^{-1} (i.p.)	[30]
Anti-inflammatory	C57BL/6 mice (DSS-induced colitis, bloody diarrhea and weight loss)	Reduction of inflammatory cytokines (IL-6, TNF-α, MCP-1 and IL-10) and suppression of NF-κB nuclear translocation	C-PC 150 mg kg^{-1} (os)	[31]
Anti-inflammatory	C57BL/6 mice (LPS and seawater-induced acute lung inflammation)	Inhibition of NF-κB/NLRP3 pathway	C-PC 100 - 400 mg kg^{-1} (i.p.)	[32]
Anti-inflammatory	C57BL/6 mice (bleomycin-induced acute pulmonary fibrosis)	Reduction of IL-6, TNF-α and MPO; reduced expression of TLR-2 pathway genes	C-PC 50 mg kg^{-1} (os)	[33]
Anti-inflammatory	Wistar rats (streptozotocin-induced) neuro-inflammation)	Upregulation of BDNF and IGF-1	C-PC 50 and 100 mg kg^{-1} (i.p.)	[34]
Anti-inflammatory	RAW 264.7 murine macrophage cell line	Suppression of NO production and iNOS	C-PC 60 - 250 μg mL^{-1}	[36]

	(LPS-stimulated macrophages)	expression, which may be associated with the attenuation of TNF-α formation and nuclear NF-κB activation		
Anti-inflammatory	Sprague-Dawley rats (LPS-induced acute lung injury)	Attenuation of expression of NF-kB; suppression of inflammatory cell infiltration, ROS generation, and pulmonary apoptosis.	C-PC<br>50 mg kg^{-1} (i.p.)	[38]
Anti-inflammatory	Primary human dermal fibroblasts (UVB-induced cell damage)	Induction of PKCα/βII/Nrf-2-mediated HO-1 pathway	C-PC<br>1-20 μg mL^{-1}	[39]
Anti-inflammatory	C57BL/6 mice (auto-immune encephalomyelitis)	Reduction of IL-17A and IL-6; gene regulation of LINGO1, NOTCH1, TNF-α, MAL, CXCL12, MOG, OLIG1, and NKX2-2	PCB-B<br>0.1-1 mg kg^{-1} (i.p.)	[40]
Anti-inflammatory	Wistar rats (acute cerebral hypoperfusion)	Decrease of (IFN)-γ, IL-6 and IL-17A.<br>Increase of MAL, NADH, BCL2A1, and BAIAP2	PCB-B<br>47 or 213 μg kg^{-1}<br>for 30 min-6 h (i.p.)	[41]
Anticancer	Human hepatocarcinoma HepG2 cell line	Increase in caspase-3 and SA-β-gal activity	C-PC<br>0.2 μg mL^{-1}	[43]
Anticancer	Human breast cancer MDA-MB-231 cell line	Decrease of cell proliferation, colony formation, cell migration; increase of cell adhesive property , anti-angiogenic effects	C-PC<br>100- 400 μg mL^{-1}	[44]
Anticancer	Sprague-Dawley rats	Inhibition of COX-2, reduction of angiogenesis	C-PC<br>200 mg kg^{-1} (os)	[45]
Anticancer	Mouse melanoma B16F10 cell line	Decrease of cell proliferation, invasion/migration; inhibition of CDK6, N-cadherin and MMP-9	C-PC<br>100- 400 μg mL^{-1}	[46]
Anticancer	A549 (human lung cancer), HT-29 (human colorectal cancer), MDA-MB-231(human breast cancer) cell lines	Antiproliferative activity, inhibitory effects on the expression of Bcl-2 and cyclin D1 proteins, G0/G1 phase arrest	C-PC<br>40 - 80 μg L^{-1}	[47-51]
Anticancer	Human breast MCF-7 cell line	Antiproliferative effect	C-PC<br>0.0625 – 2 mg ml^{-1}<br>PCB-B<br>3.125 - 100 μg ml^{-1}	[52]
Anti-diabetes	Diabetic rats	Reduction of lipids, insulin resistance, and glycemia	C-PC<br>50 mg kg^{-1} (os)<br><br>PCB-B<br>982 μg kg^{-1} (os)	[62]
Hypocholesterolemic, anti-obesity	Obese rats	Reduction of serum total cholesterol, fasting glucose serum levels and body weight	C-PC<br>250 mg Kg^{-1} day^{-1}<br>(intragastric)	[63]

Anti-obesity	Mice fed with high-fat diet	Inhibition of the increase in body mass, regulation of leptin and resistin expression, anti-inflammatory effect	C-PC<br>500 mg $kg^{-1}day^{-1}$ for 16 weeks (intragastric)	[64]
Nephroprotective	Diabetic mice	Decrease TGF- β and fibronectin expression, inhibition of oxidative stress	C-PC<br>300 mg kg^{-1} for 10 weeks (os)<br>PCB-B<br>15 mg kg^{-1} for 2 weeks (os)	[65]
Antidiabetic	KK-Ay mice	Improvement in body weight, fasting glucose levels, glycosylated proteins, glucose tolerance, total antioxidant abilities, glycogen	C-PC<br>100 mg kg^{-1} day^{-1} (os) for 3 weeks	[66]
Antidiabetic	Alloxan-induced diabetic mice	Activation of insulin signaling pathway, enhanced GK and GKRP levels in liver and pancreas	C-PC<br>100 mg kg^{-1} (os)	[67,68]
Antidiabetic	T2DM ICR mice	Increase in insulin sensitivity, cellular use of glucose; activation of AMPK, promotion of autophagy, activation of AKT	C-PC<br>200 mg kg^{-1} (os)	[71]
Antidiabetic	STZ-induced diabetic Wistar rats	Inhibition of glycation	C-PC<br>100 – 200 mg kg^{-1} (os)	[72]
Hepatoprotective	Albino rats<br>(CCl_4 or R-(+)-pulegone-treated)	Protection of liver enzymes levels (cytochrome P450, glucose-6-phosphatase and aminopyrine-N-demethylase)	C-PC<br>200 mg kg^{-1} (i.p)	[76]
Hepatoprotective	ICR Mice<br>(CCl_4-induced hepatocyte damage in vitro and in	Inhibition of lipid peroxidation and recovery of the antioxidative defense system; inhibition of TGF-1 and HGF expression.	C-PC<br>100-400 mg kg^{-1} (os) for 7 days	[77]
Hepatoprotective	Wistar rats<br>(CCl_4-treated)	Decrease of circulatory lipid peroxidation and enhancement of enzymatic and non-enzymic antioxidant concentrations (SOD, CAT, GPx, GR and GSH)	C-PC<br>75 mg kg^{-1}<br>(route of adm. not reported)	[78]
Hepatoprotective	Wistar rats<br>(galactosamine-treated)	Reduction of ALT and AST activities as well as MDA concentrations in the serum	C-PC<br>50-200 mg kg^{-1} (i.p.)	[79]
Hepatoprotective	*Ex vivo* liver from CF-1 mice<br><br>Sprague-Dawley rats<br>(T3-induced liver oxidative stress)	Inhibition of carbon phagocytosis and carbon-induced O_2 uptake.<br>Reduction of serum TNF-α, nitrite levels and activity of hepatic NOS	C-PC<br>0.1 to 0.5 mg mL^{-1}<br><br>200 mg kg^{-1} (i.p.)	[80]
Hepatoprotective	Albino rats<br>(gibberellic acid-treated)	Improvement of hepatocyte structure, antioxidant defense system and liver enzymes (CAT, SOD and GPx)	C-PC<br>200 mg kg^{-1} day^{-1} (os) for 6 weeks	[81]
Hepatoprotective	Wistar rats<br>(thioacetamide-treated)	Reduction ofAAT and AST and serum ammonia.	C-PC<br>50 mg kg^{-1} (os, twice at 24h interval)	[82]

		Decrease of tryptophan and markers of lipid peroxidation. Increase of CAT and GPx.		
Neuroprotective	Rats (focal cerebral ischemia/reperfusion (I/R) or acute brain hypoperfusion)	Reduction of the infarct volume; improvement of the exploratory activity in I/R rats; inhibition of NOX2, IFN-γ, IL-6, IL-17A, CD74, CCL12	C-PC 50 - 200 μg kg^{-1} (i.p.)	[85]
Neuroprotective	Wistar rats (focal transient brain ischaemia)	Antioxidant and mitoprotection, reduction of cerebral infarct size	PCB-B 50 - 200 μg kg^{-1} (i.p.)	[86]
Neuroprotective	Sprague-Dawley rats (laminectomy model)	Functional locomotor recovery, improvement in the fine ultrastructure of the spinal cord gray matter	C-PC 180 mg kg^{-1} (os)	[87]
Neuroprotective	Zebrafish (MPTP-induced Parkinson's disease-like pathology)	Reversed the loss of dopamine neurons and cerebral vessels, reduced the locomotor impairment, antioxidant effect	Not specified	[88]
Neuroprotective	Lewis rats (experimental autoimmune encephalomyelitis)	Restoration of the motor function, improvement in integrity of cerebral myelin sheaths, antioxidant, anti-inflammatory effects	C-PC 200 mg kg^{-1} (os)	[90]
Neuroprotective	C57BL/6 mice (autoimmune encephalomyelitis))	Anti-oxidant, anti-inflammatory and cytoprotective properties, improvement in remyelination, gliogenesis and axon-glia processes	C-PC 2 - 8 mg kg^{-1} (i.p.)	[91]
Anti-cataract	Wistar rats (selenite-treated)	Increase GSH levels and reduction of lipid peroxidation products	C-PC 100 - 200 mg kg^{-1} (i.p.)	[92]
Anti-cataract	Wistar rats (selenite-treated)	Regulation of the lens crystallin redox genes and apoptotic cascade	C-PC 200 mg kg^{-1} (i.p.)	[93]
Anti-tinnitus	SAMP8 mice (salycilate-treated)	Down-regulation of the mRNAs expression of NR2B, TNF-α, IL-1β, and COX-2 genes in the cochlea and inferior colliculus	C-PC 130 mg kg^{-1} (os)	[94]
Hearing protective	HEI-OC1 mouse auditory cell line (cisplatin-induced cytotoxicity)	Inhibition of the mitochondrial apoptotic pathway	C-PC 0.1 - 20 μg mL^{-1}	[95]
Pro-fertility (female)	B6D2F/1 mice (D-galactose-induced aging)	Antioxidant effect, reduced MDA content, improved mitochondrial distribution, reduced apoptotic mechanisms	C-PC 250, 500 mg kg^{-1} day^{-1} (os and i.p.)	[96]
Pro- fertility (female)	ICR mice (D-galactose-treated or Fed with high fat diet)	Reduction of obesity accumulation of ROS, number of early apoptotic cells, and the abnormal expression of H3K9me3 in oocytes	C-PC 500 mg kg^{-1} day^{-1} (os)	[97]

Pro-fertility (male)	GC-1 spg cell line (H_2O_2-treated)	Antioxidant activity and inhibition of necroptosis.	C-PC 0.25 - 1 mg mL^{-1}	[98]
Nephroprotective	Wistar rats (oxalate-treated)	Increase of glutathione and reduction of malondialdehyde; increase of catalase and glucose-6-phosphate dehydrogenase activity	C-PC 100 mg kg^{-1} (i.p.)	[99]
Nephroprotective	Canine kidney MDCK cell line	Reduction of the expression of phosphorylated JNK/SAPK and ERK1/2	C-PC 5 -50 mM	[100]
Nephroprotective	CD-1 mice (cisplatin induced nephrotoxicity)	Prevention of the decrease of the antioxidant enzymes	C-PC 5-30 mg kg^{-1} (i.p.)	[101]
Nephroprotective	CD-1 mice (cisplatin induced nephrotoxicity)	Attenuation of mitochondrial abnormalities	C-PC 30 mg kg^{-1} (i.p.)	[102]
Nephroprotective	C57BL/6 mice (cisplatin-induced nephrotoxicity)	Suppression of p-ERK, p-JNK, p-p38, Bax, caspase-9, and caspase-3	C-PC 50 mg kg^{-1} (i.p.)	[103]
Nephroprotective	Wistar rats (ethanol-induced nephrotoxicity)	Reduction of lipid peroxidation, SOD and CAT activities	C-PC 25, 50 mg kg^{-1} day^{-1} (i.p.) for 14 days	[104]
Nephroprotective	Mice (mercury-treated)	Prevention of alterations in antioxidant enzymes and caspase 9 activities	C-PC 0.75 – 3 mg kg^{-1} day^{-1} (os)	[105]
Anti-hypertensive	SHR/NDmcr-cp rats (metabolic syndrome model)	Improvement of endothelial function with eNOS expression enhancement	C-PC 2.5 - 10 g kg^{-1} with diet for 25 weeks	[106]
Anti-hypertensive	Wistar rats (chronic kidney disease)	Reduction of oxidative stress, NO system alterations, and endothelial dysfunction	C-PC 100 mg kg^{-1} (os)	[107]
Wound healing	Human fibroblast TIG-3-20 cell line	Modulation of GTPases, Rac 1 and Cdc 42 via PI-3K	C-PC 75 μg mL^{-1}	[108]
Photoprotective	Human keratinocytes HaCaT cell line (UVB-exposed)	Decrease in MMPs and of ROS production	C-PC 75 μg mL-1	[109]
Photo-protective	BALB/c-nu mice (UV-exposed)	Decrease of MDA levels, inflammatory mediators, expression of MMP-1, -3, -9, phosphorylation of JNK, ERK, and p38.	C-PC (200 μL of a nanodispersion containing C-PC 2.5 mg mL^{-1},prior each UVB exposure)	[111]
Antibacterial	*Propionibacterium acne*, *Staphylococcus epidermididis*, *Escherichia coli*, *Staphylococcus aureus*, *Listeria monocytogenes*, *Streptococcus iniae* and *Yersinia ruckeri*, *Aeromonas hydrophila* and *Salmonella enteritidis*	Interaction with cellular components, membrane stretching, production of pores, and cell wall	C-PC 0.5-320 μg mL^{-1}	[112-114]
Antibacterial	*Porphyromonas gingivalis*	Antioxidant activity	C-PC 1.5 – 1000 μg mL^{-1}	[115]

Antiviral	HIV-1 on TZM-bl cells	Inhibition of reverse transcriptase and ROS scavenging activity	C-PC 0.031 – 2 mg mL^{-1}	[116]

Note: CAT = catalase; C-PC = C-phycocyanin; DSS: dextran sulphate sodium; HO: heme oxygenase; LPS: lipopolysaccharide; MMP= metalloproteinase; MPO: myeloperoxidase; MPTP = 1-methyl-4-phenyl-1,2,3,6-tetrahydropyridine; iNOS= inducible nitric oxide synthase; PCB-B= phycocyanobilin B; SOD = superoxide dismutase; T3= triiodothyronine.
